# (μ-Acetato){μ-1,3-bis[2-(2-oxidobenzylideneamino)ethyl]-2-(2-oxidophenyl)-1,3-imidazolidine}dizinc(II) ethanol disolvate dihydrate

**DOI:** 10.1107/S1600536809020716

**Published:** 2009-06-06

**Authors:** Xiao-Ping Lu, Miao-Li Zhu, Li-Ping Lu

**Affiliations:** a School of Chemistry and Material Science, Shanxi Normal University, Linfen, Shanxi 041004, People’s Republic of China; b Institute of Molecular Science, Key Laboratory of Chemical Biology and Molecular Engineering of the Education Ministry, Shanxi University, Taiyuan, Shanxi 030006, People’s Republic of China

## Abstract

In the title binuclear compound, [Zn_2_(C_27_H_27_N_4_O_3_)(C_2_H_3_O_2_)]·2CH_3_CH_2_OH·2H_2_O, both Zn cations adopt distorted ZnO_3_N_2_ trigonal-bipyramidal geometries with one N atom in a axial site and one N atom in an equatorial site, arising from coordination by the *N*,*N*,*N*,*N*,*O*,*O*,*O*-hepta­dentate ligand and a bridging acetate ion. In the crystal, inter­molecular O—H⋯O hydrogen bonds link the component units into a three-dimensional network. Two short C—H⋯O contacts are also seen.

## Related literature

For further synthetic details, see: Sarma & Bailar (1955 ▶); Lu *et al.* (2007 ▶). For background information on the ligand, see: Fondo *et al.* (2002 ▶); Fondo *et al.* (2004 ▶); Prasant Kumar *et al.* (2006 ▶).
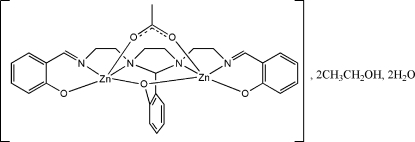


## Experimental

### 

#### Crystal data

[Zn_2_(C_27_H_27_N_4_O_3_)(C_2_H_3_O_2_)]·2CH_6_O·2H_2_O
*M* *_r_* = 773.48
Triclinic, 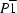

*a* = 10.140 (3) Å
*b* = 11.540 (4) Å
*c* = 16.066 (5) Å
α = 91.972 (6)°
β = 93.944 (5)°
γ = 110.833 (5)°
*V* = 1749.4 (10) Å^3^
*Z* = 2
Mo *K*α radiation
μ = 1.43 mm^−1^
*T* = 298 K
0.22 × 0.20 × 0.03 mm

#### Data collection

SMART 1K CCD diffractometer
Absorption correction: multi-scan (*SADABS*; Bruker, 2000 ▶) *T*_min_ = 0.744, *T*_max_ = 0.958
8975 measured reflections
5943 independent reflections
4785 reflections with *I* > 2σ(*I*)
*R*_int_ = 0.018

#### Refinement

*R*[*F* ^2^ > 2σ(*F* ^2^)] = 0.045
*wR*(*F* ^2^) = 0.151
*S* = 1.09
5943 reflections
437 parameters
14 restraints
H-atom parameters constrained
Δρ_max_ = 0.82 e Å^−3^
Δρ_min_ = −0.60 e Å^−3^

### 

Data collection: *SMART* (Bruker, 2000 ▶); cell refinement: *SAINT* (Bruker, 2000 ▶); data reduction: *SAINT*; program(s) used to solve structure: *SHELXS97* (Sheldrick, 2008 ▶); program(s) used to refine structure: *SHELXL97* (Sheldrick, 2008 ▶); molecular graphics: *SHELXTL/PC* (Sheldrick, 2008 ▶); software used to prepare material for publication: *SHELXTL/PC*.

## Supplementary Material

Crystal structure: contains datablocks I, global. DOI: 10.1107/S1600536809020716/hb2964sup1.cif

Structure factors: contains datablocks I. DOI: 10.1107/S1600536809020716/hb2964Isup2.hkl

Additional supplementary materials:  crystallographic information; 3D view; checkCIF report

## Figures and Tables

**Table 1:** Selected bond lengths (Å)

Zn1—O2	1.984 (2)
Zn1—O3	1.984 (3)
Zn1—O1	1.986 (3)
Zn1—N1	2.011 (3)
Zn1—N2	2.407 (3)
Zn2—O2	1.987 (2)
Zn2—O4	1.987 (3)
Zn2—O5	1.991 (3)
Zn2—N4	2.019 (3)
Zn2—N3	2.386 (3)

**Table 2:** Hydrogen-bond geometry (Å, °)

*D*—H⋯*A*	*D*—H	H⋯*A*	*D*⋯*A*	*D*—H⋯*A*
C8—H8*B*⋯O7^i^	0.97	2.41	3.259 (8)	145
C12—H12⋯O8^ii^	0.98	2.52	3.482 (5)	169
O6—H6*B*⋯O5^iii^	0.85	2.06	2.804 (4)	146
O6—H6*A*⋯O1^iii^	0.88	2.05	2.896 (4)	161
O7—H7*A*⋯O4	0.85	2.15	2.971 (8)	162
O7—H7*B*⋯O1^iv^	0.86	2.48	3.225 (9)	145
O8—H8⋯O6	0.82	1.91	2.701 (5)	163
O9—H9⋯O8^ii^	0.82	2.08	2.849 (9)	157

Symmetry codes: (i) 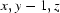; (ii) 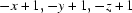; (iii) 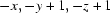; (iv) 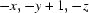.

**Fig. 1.**
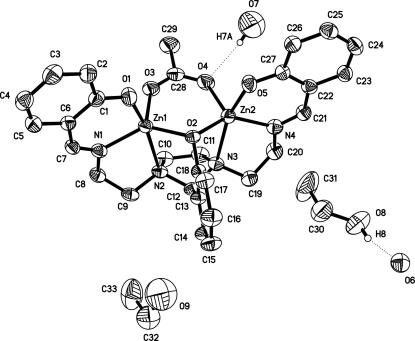
The structure of (I) with displacement ellipsoids drawn at the 30% probability level for Non-H atoms.

## supplementary crystallographic information

### Comment

In earlier studies of binuclear and tetranuclear complexes of a heptadentate Schiff base, 2-(2-hydroxyphenyl)-1,3-bis[4-(2-hydroxyphenyl)-3-azabut-3-enyl]- 1,3-imidazolidine (H_3_L), researchers reported attractive results, such as fixed atmospheric carbon dioxide (Fondo *et al.*, 2002), magnetic properties (Fondo *et al.*, 2004; Prasant Kumar *et al.*, 2006).  As part of our own work, the title complex, (I), has been syhtheszed in order to study its inhibiting activity on protein tyrosine phosphatase 1B (PTP1B), and its crystal structure is presented here.

The Zn—N and Zn—O distances and bond anges around two metal ions are in the normal range (Table 1). The molecular structure is illustrated in Fig. 1. Binuclear phenolic Schiff base complex (I) consists of Zn_2_L(OOCCH_3_) units with ethanol and water as solvates. Each zinc atom is coordinated by two N atoms and three O atoms from the heptadentate Schiff base ligand and an acetate. The intermetallic separation is 3.229 (2) Å shorter than that in Zn_2_L(OOCCH_3_).methanol.2H_2_O (Fondo *et al.*, 2002). Two zinc ions are linked by dibridges with one phenol oxygen atom of the ligand and another bidentate acetate group.

### Experimental

The heptadentate Schiff base has been prepared following a modified literature procedure (Sarma & Bailar, 1955; Lu, *et al.* 2007). The ligand was collected by filtration and recrystallized from warm ethanol. Compared IR spectroscopic data with the literature values has checked identity and purity of the ligand. The title compound (I) was synthesized as following. 0.0885 g Zn(CH_3_CH_2_O)_2_.2H_2_O was added to 0.0548 g H_3_L in 25 ml of ethanol solution with stirring, in a 1:1 molar ratio. Refluxed for 2 h, adjusted pH 8 with 1 *M* NaOH after the solution cooled to room temperature, filtered. Colourless blocks of (I) were grown from the filtrate by slow evaporation.

### Refinement

H atoms attached to C atoms and *O*(ethanol) of (I) were placed in geometrically idealized positions, with C*sp*^2^—H = 0.93, C*sp*^3^—*H*(methylene) = 0.97, C*sp*^3^—*H*(methyl) = 0.96, C*sp*^3^—H = 0.98, O—H = 0.82 Å and refined with *U*_iso_(H)=1.2*U*_eq_ or 1.5*U*_eq_(methyl, ethanol). The water H atoms were located in a difference map and refined as riding in their as-found relative positions.

### Crystal data

**Table:**

[Zn_2_(C_27_H_27_N_4_O_3_)(C_2_H_3_O_2_)]·2CH_6_O·2H_2_O	*Z* = 2
*M**_r_* = 773.48	*F*(000) = 808
Triclinic, *P*1	*D*_x_ = 1.468 Mg m^−^^3^
Hall symbol: -P 1	Mo *K*α radiation, λ = 0.71073 Å
*a* = 10.140 (3) Å	Cell parameters from 4728 reflections
*b* = 11.540 (4) Å	θ = 2.2–26.5°
*c* = 16.066 (5) Å	µ = 1.43 mm^−^^1^
α = 91.972 (6)°	*T* = 298 K
β = 93.944 (5)°	Block, colourless
γ = 110.833 (5)°	0.22 × 0.20 × 0.03 mm
*V* = 1749.4 (10) Å^3^	

### Data collection

**Table:**

SMART 1K CCD diffractometer	5943 independent reflections
Radiation source: fine-focus sealed tube	4785 reflections with *I* > 2σ(*I*)
graphite	*R*_int_ = 0.018
ω scans	θ_max_ = 25.0°, θ_min_ = 1.9°
Absorption correction: multi-scan (*SADABS*;Bruker, 2000)	*h* = −12→11
*T*_min_ = 0.744, *T*_max_ = 0.958	*k* = −13→10
8975 measured reflections	*l* = −19→17

### Refinement

**Table:**

Refinement on *F*^2^	Primary atom site location: structure-invariant direct methods
Least-squares matrix: full	Secondary atom site location: difference Fourier map
*R*[*F*^2^ > 2σ(*F*^2^)] = 0.045	Hydrogen site location: inferred from neighbouring sites
*wR*(*F*^2^) = 0.151	H-atom parameters constrained
*S* = 1.09	*w* = 1/[σ^2^(*F*_o_^2^) + (0.0929*P*)^2^ + 0.4983*P*] where *P* = (*F*_o_^2^ + 2*F*_c_^2^)/3
5943 reflections	(Δ/σ)_max_ = 0.009
437 parameters	Δρ_max_ = 0.82 e Å^−^^3^
14 restraints	Δρ_min_ = −0.60 e Å^−^^3^

### Special details

**Table:**

Geometry. All e.s.d.'s (except the e.s.d. in the dihedral angle between two l.s. planes) are estimated using the full covariance matrix. The cell e.s.d.'s are taken into account individually in the estimation of e.s.d.'s in distances, angles and torsion angles; correlations between e.s.d.'s in cell parameters are only used when they are defined by crystal symmetry. An approximate (isotropic) treatment of cell e.s.d.'s is used for estimating e.s.d.'s involving l.s. planes.
Refinement. Refinement of *F*^2^ against ALL reflections. The weighted *R*-factor *wR* and goodness of fit *S* are based on *F*^2^, conventional *R*-factors *R* are based on *F*, with *F* set to zero for negative *F*^2^. The threshold expression of *F*^2^ > σ(*F*^2^) is used only for calculating *R*-factors(gt) *etc*. and is not relevant to the choice of reflections for refinement. *R*-factors based on *F*^2^ are statistically about twice as large as those based on *F*, and *R*- factors based on ALL data will be even larger.

### Fractional atomic coordinates and isotropic or equivalent isotropic displacement parameters (Å^2^)

**Table:**

	*x*	*y*	*z*	*U*_iso_*/*U*_eq_	
Zn1	0.08495 (4)	0.22680 (4)	0.14633 (3)	0.05247 (17)	
Zn2	0.20169 (5)	0.50951 (4)	0.22548 (3)	0.05443 (17)	
N1	0.0865 (3)	0.0549 (3)	0.1233 (2)	0.0569 (8)	
N2	0.3245 (3)	0.2542 (3)	0.1953 (2)	0.0536 (7)	
N3	0.4056 (3)	0.4539 (3)	0.2503 (2)	0.0566 (8)	
N4	0.3384 (3)	0.6668 (3)	0.2863 (2)	0.0618 (8)	
C1	−0.2122 (4)	0.0545 (4)	0.1133 (2)	0.0597 (10)	
C2	−0.3590 (4)	0.0315 (4)	0.1036 (3)	0.0719 (12)	
H2	−0.3898	0.0983	0.1070	0.086*	
C3	−0.4571 (5)	−0.0861 (5)	0.0895 (3)	0.0774 (13)	
H3	−0.5528	−0.0975	0.0838	0.093*	
C4	−0.4167 (5)	−0.1883 (5)	0.0836 (3)	0.0751 (12)	
H4	−0.4839	−0.2682	0.0752	0.090*	
C5	−0.2739 (5)	−0.1683 (4)	0.0907 (3)	0.0692 (11)	
H5	−0.2455	−0.2364	0.0861	0.083*	
C6	−0.1709 (4)	−0.0504 (3)	0.1043 (2)	0.0544 (9)	
C7	−0.0231 (4)	−0.0419 (4)	0.1092 (2)	0.0566 (9)	
H7	−0.0080	−0.1163	0.1011	0.068*	
C8	0.2267 (4)	0.0441 (4)	0.1272 (3)	0.0612 (10)	
H8A	0.2710	0.0709	0.0761	0.073*	
H8B	0.2175	−0.0418	0.1332	0.073*	
C9	0.3153 (4)	0.1239 (4)	0.2005 (3)	0.0628 (10)	
H9A	0.2744	0.0921	0.2516	0.075*	
H9B	0.4098	0.1206	0.2024	0.075*	
C10	0.4315 (4)	0.3269 (4)	0.1393 (3)	0.0664 (11)	
H10A	0.5082	0.2954	0.1376	0.080*	
H10B	0.3882	0.3223	0.0829	0.080*	
C11	0.4848 (4)	0.4573 (4)	0.1762 (3)	0.0694 (11)	
H11A	0.5858	0.4853	0.1921	0.083*	
H11B	0.4670	0.5127	0.1366	0.083*	
C12	0.3722 (4)	0.3261 (4)	0.2764 (2)	0.0546 (9)	
H12	0.4596	0.3164	0.2992	0.065*	
C13	0.2647 (4)	0.2889 (3)	0.3391 (2)	0.0520 (8)	
C14	0.2967 (5)	0.2487 (4)	0.4157 (3)	0.0667 (11)	
H14	0.3854	0.2435	0.4276	0.080*	
C15	0.1989 (5)	0.2163 (5)	0.4743 (3)	0.0780 (13)	
H15	0.2220	0.1906	0.5257	0.094*	
C16	0.0674 (5)	0.2222 (5)	0.4565 (3)	0.0785 (13)	
H16	0.0010	0.1994	0.4958	0.094*	
C17	0.0326 (4)	0.2613 (4)	0.3813 (3)	0.0613 (10)	
H17	−0.0570	0.2652	0.3705	0.074*	
C18	0.1289 (4)	0.2953 (3)	0.3212 (2)	0.0477 (8)	
C19	0.4876 (4)	0.5472 (4)	0.3184 (3)	0.0697 (11)	
H19A	0.5846	0.5499	0.3242	0.084*	
H19B	0.4467	0.5235	0.3709	0.084*	
C20	0.4862 (4)	0.6744 (4)	0.2993 (3)	0.0731 (12)	
H20A	0.5351	0.7340	0.3454	0.088*	
H20B	0.5344	0.7017	0.2494	0.088*	
C21	0.3042 (4)	0.7583 (4)	0.3131 (3)	0.0612 (10)	
H21	0.3772	0.8267	0.3392	0.073*	
C22	0.1655 (4)	0.7655 (4)	0.3069 (2)	0.0566 (9)	
C23	0.1567 (5)	0.8780 (4)	0.3388 (3)	0.0633 (10)	
H23	0.2382	0.9391	0.3641	0.076*	
C24	0.0333 (5)	0.9001 (4)	0.3339 (3)	0.0696 (11)	
H24	0.0307	0.9755	0.3545	0.084*	
C25	−0.0882 (5)	0.8085 (4)	0.2975 (3)	0.0702 (11)	
H25	−0.1731	0.8225	0.2930	0.084*	
C26	−0.0841 (4)	0.6961 (4)	0.2678 (3)	0.0630 (10)	
H26	−0.1677	0.6347	0.2452	0.076*	
C27	0.0417 (4)	0.6716 (4)	0.2707 (2)	0.0537 (9)	
C28	0.1736 (4)	0.4442 (5)	0.0426 (3)	0.0613 (10)	
C29	0.1851 (7)	0.4939 (6)	−0.0427 (3)	0.0991 (18)	
H29A	0.2444	0.4622	−0.0733	0.149*	
H29B	0.2259	0.5830	−0.0374	0.149*	
H29C	0.0926	0.4684	−0.0719	0.149*	
O1	−0.1248 (3)	0.1685 (3)	0.1286 (2)	0.0772 (9)	
O2	0.0941 (2)	0.3340 (2)	0.24742 (14)	0.0472 (5)	
O3	0.1323 (4)	0.3318 (3)	0.04947 (18)	0.0733 (8)	
O4	0.2108 (4)	0.5244 (3)	0.1030 (2)	0.0794 (9)	
O5	0.0387 (3)	0.5631 (3)	0.2420 (2)	0.0691 (8)	
O6	0.2092 (3)	0.6480 (3)	0.7851 (2)	0.0703 (8)	
H6A	0.2034	0.7153	0.8090	0.105*	
H6B	0.1418	0.5853	0.7990	0.105*	
O7	0.1725 (9)	0.7596 (7)	0.0605 (5)	0.213 (4)	
H7A	0.2031	0.7019	0.0718	0.320*	
H7B	0.2001	0.7860	0.0128	0.320*	
C30	0.2098 (9)	0.5605 (8)	0.5762 (4)	0.127 (3)	
H30A	0.2234	0.4844	0.5905	0.153*	
H30B	0.1117	0.5502	0.5817	0.153*	
C31	0.2435 (14)	0.5870 (10)	0.4942 (5)	0.199 (6)	
H31A	0.1894	0.6334	0.4710	0.299*	
H31B	0.2215	0.5107	0.4609	0.299*	
H31C	0.3427	0.6350	0.4944	0.299*	
O8	0.3030 (4)	0.6641 (5)	0.6310 (3)	0.1170 (15)	
H8	0.2853	0.6509	0.6796	0.175*	
C32	0.6498 (10)	0.0359 (9)	0.4137 (6)	0.139 (3)	
H32A	0.6157	−0.0467	0.4342	0.167*	
H32B	0.7338	0.0879	0.4482	0.167*	
C33	0.6737 (12)	0.0333 (9)	0.3300 (7)	0.185 (5)	
H33A	0.6241	−0.0489	0.3049	0.278*	
H33B	0.7733	0.0557	0.3249	0.278*	
H33C	0.6402	0.0911	0.3022	0.278*	
O9	0.5470 (10)	0.0873 (7)	0.4090 (7)	0.232 (4)	
H9	0.5842	0.1632	0.4112	0.349*	

### Atomic displacement parameters (Å^2^)

**Table:**

	*U*^11^	*U*^22^	*U*^33^	*U*^12^	*U*^13^	*U*^23^
Zn1	0.0555 (3)	0.0558 (3)	0.0513 (3)	0.0263 (2)	0.00372 (19)	0.00521 (19)
Zn2	0.0532 (3)	0.0547 (3)	0.0556 (3)	0.0197 (2)	0.00390 (19)	0.00497 (19)
N1	0.0601 (19)	0.065 (2)	0.0561 (18)	0.0347 (17)	0.0064 (15)	0.0058 (15)
N2	0.0495 (17)	0.0658 (19)	0.0560 (18)	0.0310 (15)	0.0136 (14)	0.0149 (15)
N3	0.0444 (16)	0.064 (2)	0.065 (2)	0.0221 (15)	0.0084 (14)	0.0114 (15)
N4	0.0450 (17)	0.067 (2)	0.070 (2)	0.0161 (15)	0.0042 (15)	0.0061 (17)
C1	0.055 (2)	0.068 (3)	0.058 (2)	0.027 (2)	0.0006 (18)	−0.0042 (19)
C2	0.054 (2)	0.071 (3)	0.090 (3)	0.025 (2)	−0.003 (2)	−0.010 (2)
C3	0.058 (3)	0.094 (4)	0.081 (3)	0.028 (2)	0.006 (2)	−0.001 (3)
C4	0.068 (3)	0.072 (3)	0.071 (3)	0.007 (2)	0.002 (2)	0.003 (2)
C5	0.081 (3)	0.058 (2)	0.070 (3)	0.026 (2)	0.002 (2)	0.006 (2)
C6	0.061 (2)	0.055 (2)	0.049 (2)	0.0223 (18)	0.0014 (17)	0.0055 (16)
C7	0.064 (2)	0.052 (2)	0.062 (2)	0.0295 (19)	0.0052 (18)	0.0042 (17)
C8	0.059 (2)	0.066 (2)	0.070 (3)	0.036 (2)	0.0111 (19)	0.004 (2)
C9	0.057 (2)	0.079 (3)	0.069 (2)	0.043 (2)	0.0110 (19)	0.018 (2)
C10	0.053 (2)	0.086 (3)	0.069 (3)	0.032 (2)	0.0218 (19)	0.017 (2)
C11	0.050 (2)	0.079 (3)	0.083 (3)	0.023 (2)	0.022 (2)	0.020 (2)
C12	0.0420 (18)	0.074 (2)	0.052 (2)	0.0271 (17)	0.0007 (15)	0.0081 (18)
C13	0.051 (2)	0.060 (2)	0.051 (2)	0.0270 (17)	0.0050 (16)	0.0091 (16)
C14	0.060 (2)	0.090 (3)	0.059 (2)	0.039 (2)	0.0000 (19)	0.013 (2)
C15	0.090 (3)	0.101 (4)	0.052 (2)	0.044 (3)	0.007 (2)	0.023 (2)
C16	0.081 (3)	0.105 (4)	0.059 (3)	0.040 (3)	0.026 (2)	0.024 (2)
C17	0.050 (2)	0.077 (3)	0.061 (2)	0.0260 (19)	0.0149 (18)	0.010 (2)
C18	0.0456 (18)	0.055 (2)	0.0439 (18)	0.0192 (16)	0.0037 (14)	0.0028 (15)
C19	0.041 (2)	0.079 (3)	0.083 (3)	0.0179 (19)	−0.0087 (19)	−0.003 (2)
C20	0.048 (2)	0.068 (3)	0.095 (3)	0.0106 (19)	0.006 (2)	0.003 (2)
C21	0.053 (2)	0.055 (2)	0.070 (3)	0.0144 (18)	0.0028 (19)	0.0009 (19)
C22	0.059 (2)	0.051 (2)	0.059 (2)	0.0181 (17)	0.0044 (17)	0.0121 (17)
C23	0.066 (2)	0.055 (2)	0.065 (2)	0.0166 (19)	0.0029 (19)	0.0028 (18)
C24	0.086 (3)	0.055 (2)	0.074 (3)	0.031 (2)	0.020 (2)	0.010 (2)
C25	0.068 (3)	0.076 (3)	0.074 (3)	0.034 (2)	0.014 (2)	0.010 (2)
C26	0.054 (2)	0.062 (2)	0.070 (3)	0.0180 (18)	0.0047 (19)	0.0014 (19)
C27	0.054 (2)	0.053 (2)	0.055 (2)	0.0200 (17)	0.0063 (16)	0.0032 (17)
C28	0.061 (2)	0.085 (3)	0.053 (2)	0.042 (2)	0.0157 (18)	0.017 (2)
C29	0.140 (5)	0.107 (4)	0.064 (3)	0.057 (4)	0.021 (3)	0.029 (3)
O1	0.0533 (16)	0.0614 (18)	0.116 (3)	0.0245 (14)	−0.0111 (16)	−0.0170 (17)
O2	0.0396 (12)	0.0561 (14)	0.0486 (13)	0.0202 (10)	0.0035 (10)	0.0065 (10)
O3	0.097 (2)	0.075 (2)	0.0534 (16)	0.0353 (17)	0.0087 (15)	0.0175 (14)
O4	0.110 (3)	0.076 (2)	0.0631 (19)	0.0431 (19)	0.0184 (17)	0.0240 (16)
O5	0.0545 (15)	0.0592 (17)	0.090 (2)	0.0205 (13)	−0.0054 (14)	−0.0136 (15)
O6	0.0563 (16)	0.0773 (19)	0.080 (2)	0.0279 (14)	0.0070 (14)	0.0029 (15)
O7	0.290 (10)	0.149 (6)	0.199 (8)	0.087 (6)	−0.036 (7)	0.008 (5)
C30	0.158 (7)	0.158 (7)	0.089 (4)	0.090 (6)	−0.008 (4)	−0.005 (4)
C31	0.296 (15)	0.171 (9)	0.074 (5)	0.011 (9)	0.045 (6)	−0.008 (5)
O8	0.094 (3)	0.174 (4)	0.076 (2)	0.044 (3)	−0.002 (2)	−0.012 (3)
C32	0.139 (3)	0.139 (3)	0.139 (3)	0.0500 (13)	0.0112 (10)	0.0098 (10)
C33	0.205 (11)	0.136 (7)	0.158 (9)	−0.014 (7)	0.053 (8)	−0.001 (6)
O9	0.232 (4)	0.232 (4)	0.233 (4)	0.0837 (17)	0.0205 (11)	0.0152 (11)

### Geometric parameters (Å, °)

**Table:**

Zn1—O2	1.984 (2)	C15—H15	0.9300
Zn1—O3	1.984 (3)	C16—C17	1.370 (6)
Zn1—O1	1.986 (3)	C16—H16	0.9300
Zn1—N1	2.011 (3)	C17—C18	1.387 (5)
Zn1—N2	2.407 (3)	C17—H17	0.9300
Zn2—O2	1.987 (2)	C18—O2	1.350 (4)
Zn2—O4	1.987 (3)	C19—C20	1.515 (7)
Zn2—O5	1.991 (3)	C19—H19A	0.9700
Zn2—N4	2.019 (3)	C19—H19B	0.9700
Zn2—N3	2.386 (3)	C20—H20A	0.9700
N1—C7	1.265 (5)	C20—H20B	0.9700
N1—C8	1.468 (5)	C21—C22	1.435 (6)
N2—C12	1.474 (5)	C21—H21	0.9300
N2—C9	1.479 (5)	C22—C27	1.406 (5)
N2—C10	1.493 (5)	C22—C23	1.414 (6)
N3—C12	1.474 (5)	C23—C24	1.361 (6)
N3—C11	1.476 (5)	C23—H23	0.9300
N3—C19	1.485 (5)	C24—C25	1.381 (7)
N4—C21	1.292 (5)	C24—H24	0.9300
N4—C20	1.469 (5)	C25—C26	1.382 (6)
C1—O1	1.302 (5)	C25—H25	0.9300
C1—C2	1.412 (6)	C26—C27	1.400 (6)
C1—C6	1.421 (5)	C26—H26	0.9300
C2—C3	1.368 (7)	C27—O5	1.308 (5)
C2—H2	0.9300	C28—O3	1.225 (5)
C3—C4	1.381 (7)	C28—O4	1.256 (5)
C3—H3	0.9300	C28—C29	1.500 (6)
C4—C5	1.378 (7)	C29—H29A	0.9600
C4—H4	0.9300	C29—H29B	0.9600
C5—C6	1.389 (6)	C29—H29C	0.9600
C5—H5	0.9300	O6—H6A	0.8774
C6—C7	1.463 (5)	O6—H6B	0.8491
C7—H7	0.9300	O7—H7A	0.8487
C8—C9	1.495 (6)	O7—H7B	0.8620
C8—H8A	0.9700	C30—C31	1.400 (10)
C8—H8B	0.9700	C30—O8	1.452 (9)
C9—H9A	0.9700	C30—H30A	0.9700
C9—H9B	0.9700	C30—H30B	0.9700
C10—C11	1.491 (7)	C31—H31A	0.9600
C10—H10A	0.9700	C31—H31B	0.9600
C10—H10B	0.9700	C31—H31C	0.9600
C11—H11A	0.9700	O8—H8	0.8200
C11—H11B	0.9700	C32—O9	1.370 (11)
C12—C13	1.496 (5)	C32—C33	1.383 (12)
C12—H12	0.9800	C32—H32A	0.9700
C13—C14	1.387 (5)	C32—H32B	0.9700
C13—C18	1.415 (5)	C33—H33A	0.9600
C14—C15	1.378 (6)	C33—H33B	0.9600
C14—H14	0.9300	C33—H33C	0.9600
C15—C16	1.370 (7)	O9—H9	0.8200
			
O2—Zn1—O3	108.98 (12)	C15—C14—C13	120.9 (4)
O2—Zn1—O1	93.56 (11)	C15—C14—H14	119.5
O3—Zn1—O1	98.80 (15)	C13—C14—H14	119.5
O2—Zn1—N1	135.96 (11)	C16—C15—C14	119.7 (4)
O3—Zn1—N1	113.08 (13)	C16—C15—H15	120.1
O1—Zn1—N1	92.47 (13)	C14—C15—H15	120.1
O2—Zn1—N2	84.15 (10)	C17—C16—C15	120.7 (4)
O3—Zn1—N2	96.71 (12)	C17—C16—H16	119.6
O1—Zn1—N2	164.21 (13)	C15—C16—H16	119.6
N1—Zn1—N2	78.67 (12)	C16—C17—C18	121.0 (4)
O2—Zn2—O4	109.20 (12)	C16—C17—H17	119.5
O2—Zn2—O5	93.41 (11)	C18—C17—H17	119.5
O4—Zn2—O5	99.82 (14)	O2—C18—C17	120.8 (3)
O2—Zn2—N4	139.65 (12)	O2—C18—C13	120.7 (3)
O4—Zn2—N4	109.35 (14)	C17—C18—C13	118.6 (3)
O5—Zn2—N4	91.40 (13)	N3—C19—C20	110.2 (4)
O2—Zn2—N3	84.67 (10)	N3—C19—H19A	109.6
O4—Zn2—N3	97.01 (13)	C20—C19—H19A	109.6
O5—Zn2—N3	162.73 (13)	N3—C19—H19B	109.6
N4—Zn2—N3	79.32 (13)	C20—C19—H19B	109.6
C7—N1—C8	119.3 (3)	H19A—C19—H19B	108.1
C7—N1—Zn1	124.7 (3)	N4—C20—C19	108.8 (3)
C8—N1—Zn1	115.9 (3)	N4—C20—H20A	109.9
C12—N2—C9	112.3 (3)	C19—C20—H20A	109.9
C12—N2—C10	103.0 (3)	N4—C20—H20B	109.9
C9—N2—C10	113.8 (3)	C19—C20—H20B	109.9
C12—N2—Zn1	113.4 (2)	H20A—C20—H20B	108.3
C9—N2—Zn1	101.4 (2)	N4—C21—C22	126.8 (4)
C10—N2—Zn1	113.3 (2)	N4—C21—H21	116.6
C12—N3—C11	103.6 (3)	C22—C21—H21	116.6
C12—N3—C19	111.6 (3)	C27—C22—C23	118.9 (4)
C11—N3—C19	113.1 (3)	C27—C22—C21	125.2 (4)
C12—N3—Zn2	113.5 (2)	C23—C22—C21	115.9 (4)
C11—N3—Zn2	114.1 (2)	C24—C23—C22	122.4 (4)
C19—N3—Zn2	101.3 (2)	C24—C23—H23	118.8
C21—N4—C20	119.6 (4)	C22—C23—H23	118.8
C21—N4—Zn2	124.6 (3)	C23—C24—C25	118.7 (4)
C20—N4—Zn2	115.8 (3)	C23—C24—H24	120.6
O1—C1—C2	118.7 (4)	C25—C24—H24	120.6
O1—C1—C6	124.7 (4)	C24—C25—C26	120.4 (4)
C2—C1—C6	116.6 (4)	C24—C25—H25	119.8
C3—C2—C1	121.9 (4)	C26—C25—H25	119.8
C3—C2—H2	119.1	C25—C26—C27	122.1 (4)
C1—C2—H2	119.1	C25—C26—H26	118.9
C2—C3—C4	121.3 (4)	C27—C26—H26	118.9
C2—C3—H3	119.4	O5—C27—C26	119.3 (4)
C4—C3—H3	119.4	O5—C27—C22	123.3 (3)
C5—C4—C3	118.1 (4)	C26—C27—C22	117.4 (4)
C5—C4—H4	121.0	O3—C28—O4	124.7 (4)
C3—C4—H4	121.0	O3—C28—C29	119.7 (4)
C4—C5—C6	122.4 (4)	O4—C28—C29	115.7 (4)
C4—C5—H5	118.8	C28—C29—H29A	109.5
C6—C5—H5	118.8	C28—C29—H29B	109.5
C5—C6—C1	119.6 (4)	H29A—C29—H29B	109.5
C5—C6—C7	116.9 (4)	C28—C29—H29C	109.5
C1—C6—C7	123.4 (3)	H29A—C29—H29C	109.5
N1—C7—C6	127.4 (3)	H29B—C29—H29C	109.5
N1—C7—H7	116.3	C1—O1—Zn1	127.1 (3)
C6—C7—H7	116.3	C18—O2—Zn1	116.5 (2)
N1—C8—C9	108.4 (3)	C18—O2—Zn2	116.3 (2)
N1—C8—H8A	110.0	Zn1—O2—Zn2	108.80 (11)
C9—C8—H8A	110.0	C28—O3—Zn1	133.2 (3)
N1—C8—H8B	110.0	C28—O4—Zn2	131.8 (3)
C9—C8—H8B	110.0	C27—O5—Zn2	128.0 (2)
H8A—C8—H8B	108.4	H6A—O6—H6B	108.8
N2—C9—C8	111.1 (3)	H7A—O7—H7B	108.3
N2—C9—H9A	109.4	C31—C30—O8	107.5 (8)
C8—C9—H9A	109.4	C31—C30—H30A	110.2
N2—C9—H9B	109.4	O8—C30—H30A	110.2
C8—C9—H9B	109.4	C31—C30—H30B	110.2
H9A—C9—H9B	108.0	O8—C30—H30B	110.2
C11—C10—N2	105.4 (3)	H30A—C30—H30B	108.5
C11—C10—H10A	110.7	C30—C31—H31A	109.5
N2—C10—H10A	110.7	C30—C31—H31B	109.5
C11—C10—H10B	110.7	H31A—C31—H31B	109.5
N2—C10—H10B	110.7	C30—C31—H31C	109.5
H10A—C10—H10B	108.8	H31A—C31—H31C	109.5
N3—C11—C10	105.2 (3)	H31B—C31—H31C	109.5
N3—C11—H11A	110.7	C30—O8—H8	109.5
C10—C11—H11A	110.7	O9—C32—C33	99.1 (10)
N3—C11—H11B	110.7	O9—C32—H32A	111.9
C10—C11—H11B	110.7	C33—C32—H32A	111.9
H11A—C11—H11B	108.8	O9—C32—H32B	111.9
N3—C12—N2	101.1 (3)	C33—C32—H32B	112.0
N3—C12—C13	114.4 (3)	H32A—C32—H32B	109.6
N2—C12—C13	113.5 (3)	C32—C33—H33A	109.5
N3—C12—H12	109.2	C32—C33—H33B	109.5
N2—C12—H12	109.2	H33A—C33—H33B	109.5
C13—C12—H12	109.2	C32—C33—H33C	109.5
C14—C13—C18	119.1 (3)	H33A—C33—H33C	109.5
C14—C13—C12	120.7 (3)	H33B—C33—H33C	109.5
C18—C13—C12	120.2 (3)	C32—O9—H9	109.5
			
O2—Zn1—N1—C7	94.1 (3)	N3—C12—C13—C14	−122.5 (4)
O3—Zn1—N1—C7	−104.3 (3)	N2—C12—C13—C14	122.1 (4)
O1—Zn1—N1—C7	−3.6 (3)	N3—C12—C13—C18	57.0 (5)
N2—Zn1—N1—C7	163.2 (3)	N2—C12—C13—C18	−58.4 (5)
O2—Zn1—N1—C8	−83.2 (3)	C18—C13—C14—C15	−0.7 (7)
O3—Zn1—N1—C8	78.5 (3)	C12—C13—C14—C15	178.9 (4)
O1—Zn1—N1—C8	179.1 (3)	C13—C14—C15—C16	0.9 (8)
N2—Zn1—N1—C8	−14.1 (3)	C14—C15—C16—C17	−0.8 (8)
O2—Zn1—N2—C12	3.6 (2)	C15—C16—C17—C18	0.4 (8)
O3—Zn1—N2—C12	112.1 (3)	C16—C17—C18—O2	−179.9 (4)
O1—Zn1—N2—C12	−78.7 (5)	C16—C17—C18—C13	−0.1 (6)
N1—Zn1—N2—C12	−135.6 (3)	C14—C13—C18—O2	180.0 (4)
O2—Zn1—N2—C9	124.3 (2)	C12—C13—C18—O2	0.4 (5)
O3—Zn1—N2—C9	−127.2 (2)	C14—C13—C18—C17	0.3 (6)
O1—Zn1—N2—C9	41.9 (5)	C12—C13—C18—C17	−179.3 (4)
N1—Zn1—N2—C9	−15.0 (2)	C12—N3—C19—C20	−164.7 (3)
O2—Zn1—N2—C10	−113.3 (3)	C11—N3—C19—C20	78.9 (4)
O3—Zn1—N2—C10	−4.8 (3)	Zn2—N3—C19—C20	−43.7 (4)
O1—Zn1—N2—C10	164.3 (4)	C21—N4—C20—C19	142.6 (4)
N1—Zn1—N2—C10	107.5 (3)	Zn2—N4—C20—C19	−37.8 (5)
O2—Zn2—N3—C12	−4.4 (2)	N3—C19—C20—N4	56.2 (5)
O4—Zn2—N3—C12	−113.1 (3)	C20—N4—C21—C22	−179.0 (4)
O5—Zn2—N3—C12	79.9 (5)	Zn2—N4—C21—C22	1.4 (6)
N4—Zn2—N3—C12	138.4 (3)	N4—C21—C22—C27	0.7 (7)
O2—Zn2—N3—C11	114.0 (3)	N4—C21—C22—C23	−177.9 (4)
O4—Zn2—N3—C11	5.3 (3)	C27—C22—C23—C24	−1.7 (6)
O5—Zn2—N3—C11	−161.7 (4)	C21—C22—C23—C24	177.0 (4)
N4—Zn2—N3—C11	−103.2 (3)	C22—C23—C24—C25	1.1 (7)
O2—Zn2—N3—C19	−124.1 (3)	C23—C24—C25—C26	0.8 (7)
O4—Zn2—N3—C19	127.2 (3)	C24—C25—C26—C27	−2.0 (7)
O5—Zn2—N3—C19	−39.8 (5)	C25—C26—C27—O5	179.6 (4)
N4—Zn2—N3—C19	18.7 (3)	C25—C26—C27—C22	1.3 (6)
O2—Zn2—N4—C21	−101.8 (4)	C23—C22—C27—O5	−177.7 (4)
O4—Zn2—N4—C21	96.1 (4)	C21—C22—C27—O5	3.6 (6)
O5—Zn2—N4—C21	−4.9 (4)	C23—C22—C27—C26	0.5 (6)
N3—Zn2—N4—C21	−170.2 (4)	C21—C22—C27—C26	−178.1 (4)
O2—Zn2—N4—C20	78.5 (3)	C2—C1—O1—Zn1	179.1 (3)
O4—Zn2—N4—C20	−83.6 (3)	C6—C1—O1—Zn1	−1.6 (6)
O5—Zn2—N4—C20	175.5 (3)	O2—Zn1—O1—C1	−132.8 (4)
N3—Zn2—N4—C20	10.2 (3)	O3—Zn1—O1—C1	117.4 (4)
O1—C1—C2—C3	−178.3 (5)	N1—Zn1—O1—C1	3.6 (4)
C6—C1—C2—C3	2.4 (7)	N2—Zn1—O1—C1	−51.7 (7)
C1—C2—C3—C4	−0.3 (8)	C17—C18—O2—Zn1	−114.9 (3)
C2—C3—C4—C5	−1.3 (7)	C13—C18—O2—Zn1	65.3 (4)
C3—C4—C5—C6	0.8 (7)	C17—C18—O2—Zn2	114.7 (3)
C4—C5—C6—C1	1.3 (6)	C13—C18—O2—Zn2	−65.0 (4)
C4—C5—C6—C7	−179.0 (4)	O3—Zn1—O2—C18	−150.8 (2)
O1—C1—C6—C5	177.9 (4)	O1—Zn1—O2—C18	108.6 (2)
C2—C1—C6—C5	−2.8 (6)	N1—Zn1—O2—C18	11.3 (3)
O1—C1—C6—C7	−1.7 (6)	N2—Zn1—O2—C18	−55.7 (2)
C2—C1—C6—C7	177.6 (4)	O3—Zn1—O2—Zn2	−17.05 (15)
C8—N1—C7—C6	179.0 (4)	O1—Zn1—O2—Zn2	−117.62 (13)
Zn1—N1—C7—C6	1.8 (6)	N1—Zn1—O2—Zn2	145.11 (15)
C5—C6—C7—N1	−178.0 (4)	N2—Zn1—O2—Zn2	78.06 (11)
C1—C6—C7—N1	1.6 (6)	O4—Zn2—O2—C18	151.3 (2)
C7—N1—C8—C9	−135.9 (4)	O5—Zn2—O2—C18	−107.1 (2)
Zn1—N1—C8—C9	41.5 (4)	N4—Zn2—O2—C18	−10.9 (3)
C12—N2—C9—C8	162.5 (3)	N3—Zn2—O2—C18	55.7 (2)
C10—N2—C9—C8	−81.0 (4)	O4—Zn2—O2—Zn1	17.41 (15)
Zn1—N2—C9—C8	41.1 (3)	O5—Zn2—O2—Zn1	119.05 (13)
N1—C8—C9—N2	−56.5 (4)	N4—Zn2—O2—Zn1	−144.75 (16)
C12—N2—C10—C11	−26.8 (4)	N3—Zn2—O2—Zn1	−78.17 (12)
C9—N2—C10—C11	−148.6 (3)	O4—C28—O3—Zn1	7.9 (7)
Zn1—N2—C10—C11	96.2 (3)	C29—C28—O3—Zn1	−173.5 (4)
C12—N3—C11—C10	28.0 (4)	O2—Zn1—O3—C28	6.1 (4)
C19—N3—C11—C10	149.0 (3)	O1—Zn1—O3—C28	103.0 (4)
Zn2—N3—C11—C10	−95.8 (3)	N1—Zn1—O3—C28	−160.5 (4)
N2—C10—C11—N3	−0.7 (4)	N2—Zn1—O3—C28	−80.0 (4)
C11—N3—C12—N2	−44.8 (3)	O3—C28—O4—Zn2	−7.1 (7)
C19—N3—C12—N2	−166.8 (3)	C29—C28—O4—Zn2	174.2 (4)
Zn2—N3—C12—N2	79.5 (3)	O2—Zn2—O4—C28	−7.3 (4)
C11—N3—C12—C13	−167.2 (3)	O5—Zn2—O4—C28	−104.4 (4)
C19—N3—C12—C13	70.8 (4)	N4—Zn2—O4—C28	160.6 (4)
Zn2—N3—C12—C13	−42.9 (4)	N3—Zn2—O4—C28	79.5 (4)
C9—N2—C12—N3	166.8 (3)	C26—C27—O5—Zn2	172.0 (3)
C10—N2—C12—N3	43.9 (3)	C22—C27—O5—Zn2	−9.8 (6)
Zn1—N2—C12—N3	−79.0 (3)	O2—Zn2—O5—C27	149.1 (3)
C9—N2—C12—C13	−70.2 (4)	O4—Zn2—O5—C27	−100.7 (3)
C10—N2—C12—C13	166.9 (3)	N4—Zn2—O5—C27	9.2 (3)
Zn1—N2—C12—C13	44.1 (4)	N3—Zn2—O5—C27	66.1 (6)

### Hydrogen-bond geometry (Å, °)

**Table:**

*D*—H···*A*	*D*—H	H···*A*	*D*···*A*	*D*—H···*A*
C8—H8B···O7^i^	0.97	2.41	3.259 (8)	145
C12—H12···O8^ii^	0.98	2.52	3.482 (5)	169
O6—H6B···O5^iii^	0.85	2.06	2.804 (4)	146
O6—H6A···O1^iii^	0.88	2.05	2.896 (4)	161
O7—H7A···O4	0.85	2.15	2.971 (8)	162
O7—H7B···O1^iv^	0.86	2.48	3.225 (9)	145
O8—H8···O6	0.82	1.91	2.701 (5)	163
O9—H9···O8^ii^	0.82	2.08	2.849 (9)	157

Symmetry codes: (i) *x*, *y*−1, *z*; (ii) −*x*+1, −*y*+1, −*z*+1; (iii) −*x*, −*y*+1, −*z*+1; (iv) −*x*, −*y*+1, −*z*.
